# Pilot Evaluation of a Deep Learning Model for Nasogastric Tube Verification on Chest Radiographs: A Single-Center Retrospective Study

**DOI:** 10.3390/tomography11120140

**Published:** 2025-12-15

**Authors:** Sang Won Park, Doohee Lee, Jae Eun Song, Yoon Kim, Hyun-Soo Choi, Seung-Joon Lee, Woo Jin Kim, Kyoung Min Moon, Oh Beom Kwon

**Affiliations:** 1 Department of Next Generation Information Center, Kangwon National University Hospital, Chuncheon 24289, Republic of Korea; chicwon229@kangwon.ac.kr; 2 Department of Research and Development, ZIOVISION Co., Ltd., Chuncheon 24341, Republic of Korea; getback9@gmail.com (D.L.); yooni@kangwon.ac.kr (Y.K.); choi.hyunsoo@seoultech.ac.kr (H.-S.C.); 3 Department of Computer Science and Engineering, College of IT, Kangwon National University, Chuncheon 24341, Republic of Korea; 4 Department of Radiology, Kangwon National University Hospital, Chuncheon 24289, Republic of Korea; ccbigsko@naver.com; 5 Department of Computer Science and Engineering, Seoul National University of Science and Technology, Seoul 01811, Republic of Korea; 6 Division of Pulmonology, Department of Internal Medicine, Kangwon National University Hospital, Chuncheon 24289, Republic of Korea; medfman@kangwon.ac.kr (S.-J.L.); pulmo2@kangwon.ac.kr (W.J.K.); 7 Division of Pulmonary and Allergy Medicine, Department of Internal Medicine, Chung-Ang University Hospital, Seoul 06973, Republic of Korea; pulmogicu@gmail.com

**Keywords:** deep learning model, nasogastric tube, real-world validation

## Abstract

Background: Accurate confirmation of nasogastric (NG) tubes is essential for patient safety, but delays and variability in interpretation remain common in clinical practice. Deep learning (DL) models have shown potential for assisting in this task, but real-world performance, particularly in detecting malpositioned tubes, remains insufficiently characterized. Methods: We conducted a pilot evaluation of a previously developed DL model using 135 chest radiographs from Kangwon National University Hospital. Expert physicians established the reference standard. Model performance was assessed and receiver operating characteristic (ROC) curve and precision recall curve (PRC) analyses were performed. Differences between correctly classified and misclassified cases were examined using Wilcoxon rank-sum and Fisher’s exact tests to explore potential clinical or radiographic contributors to model failure. Results: The model correctly classified 129 of 135 cases. The sensitivity was 96.1% (95% confidence interval (CI): 92.2–98.9%), specificity was 85.7% (95% CI: 42.2–97.7%), positive predictive value (PPV) was 99.2% (95% CI: 96.1–99.9%), negative predictive value (NPV) was 54.5% (95% CI: 25.4–80.8%), balanced accuracy was 90.8%, and F1-score was 0.976. The area under the ROC curve was 0.970 (95% CI: 0.929–1.000) and that under the PRC was 0.727 (95% CI: 0.289–1.000), reflecting substantial uncertainty related to the very small number of incomplete cases (*n* = 6). No statistically significant differences in clinical or radiographic characteristics were observed between correctly classified and misclassified cases. Conclusions: The DL model performed well in identifying correctly positioned NG tubes but demonstrated limited and unstable performance for detecting incomplete placements. Given the safety implications of misclassification, the model should be used only as an assistive tool with mandatory physician oversight. Larger, multi-center studies with greater representation of incomplete cases are required to obtain more reliable estimates and support safe clinical implementation.

## Simple Summary

We evaluated a deep learning (DL) model for assessing nasogastric (NG) tube placement on chest radiographs using institutional clinical data. The model demonstrated high accuracy in identifying correctly positioned tubes but showed reduced performance for incomplete placements, reflecting the small number of such cases. Although the system may facilitate more efficient confirmation of NG tube position, clinical oversight remains essential to prevent safety-critical errors. The findings indicate a need for larger and more balanced datasets to enhance performance and support broader clinical applicability.

## 1. Introduction

Nasogastric (NG) tubes are frequently inserted into patients with dysphagia or those who are endotracheally intubated. An NG tube is inserted through the nostril, passes the nasopharynx and esophagus, and reaches the stomach. Ideally, it should be positioned approximately 10 cm below the gastroesophageal junction, but in clinical practice, bronchial misplacement occurs in 2–4% of NG tube insertions [1], which may lead to pneumonia or pneumothorax [2], or even death. Therefore, confirming NG tube placement is crucial in clinical practice. However, most existing deep learning (DL) approaches in this domain have focused solely on binary classification of NG tube position without providing explicit spatial localization or trajectory visualization for the clinician [3,4].

Chest X-ray is the standard method for confirming NG tube placement. However, interpreting chest radiographs can be challenging for junior physicians [3], and difficulties may persist even when standard criteria are applied. Furthermore, radiographic confirmation delays feeding by more than 2 h in approximately 51% of cases [2]. Thus, there is an unmet clinical need for a system that assists in chest radiograph interpretation and shortens confirmation time, thereby enabling enteral feeding.

Recently, DL methods have shown remarkable performance in medical image analysis, including detection, segmentation, and classification tasks [5]. These models have also been applied to identify tubes and lines on chest radiographs, yielding promising results [6,7]. Our previously developed dual-stage model uniquely integrates a segmentation and a classification module, thereby enabling both accurate placement assessment and interpretable visualization of the NG tube trajectory [8]. This model achieved an area under the curve (AUC) of 99.72% for classification, and not only classifies cases as complete (safe for feeding) or incomplete (unsafe for feeding) but also provides a detailed spatial representation of the NG tube path, assisting physicians in verifying its placement [8].

Albumin is the major component of colloid oncotic pressure, but hypoalbuminemia reduces this pressure, resulting in pulmonary edema [9]. Similarly, renal dysfunction or patients with cardiomegaly can contribute to fluid overload and pulmonary congestion. Pulmonary edema increases the radiographic brightness of the lung fields, thereby reducing the contrast between radiopaque medical devices and surrounding tissues. This effect can make it more challenging to detect the tip of NG tubes. A previous study showed that high body mass index (BMI) and male sex were associated with a risk of insufficient visibility of an NG tube on an X-ray. This is because X-ray penetration is reduced in patients with higher BMI, and males typically exhibit higher BMI than females [10]. Since albumin, renal function, cardiomegaly, BMI, and sex may affect the visibility of this NG tube, these variables were considered in our analysis.

In this study, we applied the previously developed DL model in a real-world clinical setting. In intensive-care and high-volume hospital settings, delays in radiographic confirmation often postpone enteral feeding and reduce workflow efficiency [1,2]; therefore, an artificial intelligence (AI)-assisted system validated under real-world conditions is urgently needed. The aims of this study were (a) to validate the model’s performance and its agreement with physicians and (b) to evaluate the model’s performance under conditions that may affect X-ray visibility. Furthermore, by providing visual feedback of the NG tube trajectory, the model enhances interpretability and clinician trust—critical factors for successful AI adoption in clinical radiographic practice.

## 2. Materials and Methods

### 2.1. Study Population and Data Collection

In this retrospective pilot study, a total of 135 consecutive cases of chest radiographs with NG tubes were collected from Kangwon National University Hospital between March and September 2025. The chest radiograph images used in this study were acquired using digital radiography systems from Simens (FLUOROSPOT Compact FD, Munich, Germany) and Samsung (GM85, Seoul, Republic of Korea). The imaging parameters for the FLUOROSPOT Compact FD were 77 kV and 2 mAs, while those for the GM85 were 85 kV and 2 mAs. All radiographs were obtained as portable examinations for patients admitted to the hospital wards.

A radiologist (S.J.E., 8 years of clinical experience) and a pulmonologist (O.B.K., 12 years of clinical experience) independently reviewed all cases and classified them as either complete or incomplete in a blinded manner. Cases were defined as complete if feeding through the NG tube was considered safe, and incomplete otherwise. The time taken by the radiologist and the pulmonologist to confirm the chest radiographs with the aid of the DL model was measured. The DL model generated a probability score between 0 and 1 for each case, with higher values indicating a higher likelihood of correct (complete) NG tube placement. Based on our previous model development study, which demonstrated an AUC of 99.72%, a strict probability threshold of 0.90 was established [8]. This conservative threshold was selected to maximize specificity and minimize false-positive classifications (i.e., identifying a misplaced tube as safe), thereby prioritizing patient safety in clinical practice. Scores greater than 0.90 were classified as complete, whereas those ≤0.90 were classified as incomplete. Agreement among the radiologist, the pulmonologist, and the DL model was assessed using Cohen’s κ, PABAK, and Gwet’s AC_1_ coefficients.

Patients’ demographic data including age, sex, and BMI and laboratory data including white blood cell (WBC), hemoglobin, platelet, blood urea nitrogen (BUN), creatinine, estimated glomerular filtration rate (eGFR), and cardiac–thoracic (CT) ratio were collected. Because devices such as central lines, pacemakers, or artificial valves could affect the analysis, the presence of these devices was additionally recorded. To explore factors associated with model misclassification, cases were divided into two groups: correctly classified and misclassified. Because the number of misclassified cases was extremely small, the Wilcoxon rank-sum test was used to compare continuous variables and Fisher’s exact test was used to compare categorical variables.

In clinical settings, after NG tube insertion, patients are sent to take chest radiographs and nurses contact the physicians to confirm the images. The time intervals between NG tube insertion and chest radiograph acquisition were extracted from the electronic medical records (EMR). Similarly, the time intervals between radiograph acquisition and the physician’s confirmation of the findings were also obtained from the EMR. The time between X-ray acquisition and physician confirmation from the EMR and the time taken by the physicians with the aid of the deep learning model were measured.

### 2.2. Model Explanation

**Figure 1:**
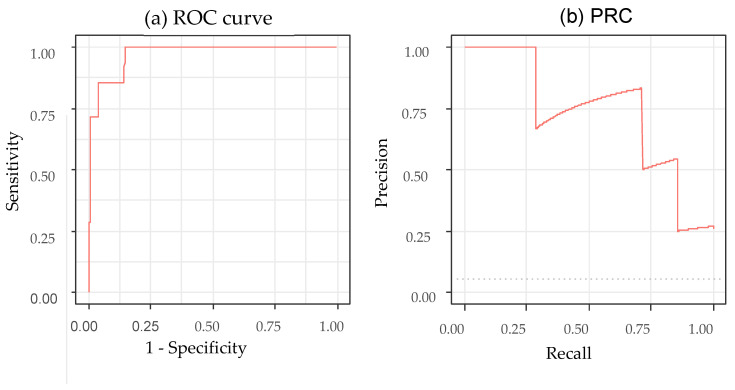
Receiver operating characteristic curve and precision recall curve for the deep learning model. ROC, receiver operating characteristic; PRC, precision recall curve.

We utilized the DL-based dual-stage model developed and comprehensively characterized in our previous study [8]. This comprehensive workflow, which consists of segmentation, concatenation, and classification, is fully illustrated in Figure 1 of our previous study [8].

This architecture comprises segmentation and classification stages. Specifically, nnU-Net (version 2.5.1) was employed for the segmentation stage, which precisely delineates the location and trajectory of the NG tube within the chest radiograph. This module achieved robust segmentation performance, achieving a Dice Similarity Coefficient of 65.35% and a Jaccard coefficient of 57.49% on the dedicated testing set. These metrics are significant given the fine, linear structure of the tube.

The classification stage is critically informed by the segmentation output through a concatenation step. The input for the classification model is created by combining the original X-ray image with the segmented line mask, which encodes the tube’s shape and path information, and results in an enhanced, multi-channel input. This novel approach imparts essential shape awareness to the subsequent classification module. The enhanced input is then fed into the classification model, utilizing a ResNet50 architecture pre-trained with MedCLIP [11] for the final prediction. This model predicts the probability of the NG tube positioning being classified as ‘complete’ or ‘incomplete’ (malposition).

This model was trained on 1799 chest radiographs collected from three major institutions (Hallym University Sacred Heart Hospital, Gangneung Asan Hospital, and Kangwon National University Hospital). All networks and experimental settings were implemented using the PyTorch framework (version 2.4.1.) under Python 3.11.9, with CUDA 12.1 and cuDNN 9 libraries. The prototype is accessible at https://ngtube.ziovision.ai (accessed on 25 October 2025). The model parameters were fixed, and no additional training or fine-tuning was performed. This study represents an internal retrospective evaluation using a fully pre-trained model. Statistical analysis was performed using R software, version 4.4.2. (R Foundation for Statistical Computing, Vienna, Austria).

## 3. Results

### 3.1. Basic Characteristics of the Cases

The basic characteristics of the patients are shown in Table 1. No statistically significant differences were observed between correctly classified and misclassified cases across all evaluated clinical and radiographic variables.

### 3.2. Time Intervals Between NG Tube Insertion and Physician Confirmation

The time intervals between NG tube insertion and physician confirmation are shown in Supplementary Table S1. The mean time interval between NG tube insertion and chest radiograph acquisition was 31.5 min, and that between radiograph acquisition and physician confirmation was 75.6 min in clinical practice. The mean time spent by the radiologist and pulmonologist to confirm NG tube placement with the aid of the DL model was 1.21 and 1.33 s, respectively. These reading times represent pure interpretation time under controlled conditions rather than real clinical workflow latency, and therefore were not used for primary performance evaluation.

### 3.3. Classification of Cases and Reliability Analysis

The classification outcomes of the DL model compared with physician-determined reference standards are summarized in Table 2. Among 135 cases, the two physicians identically classified 128 cases as complete and 7 as incomplete, whereas the DL model classified 124 cases as complete and 11 as incomplete. A total of six cases were misclassified, among which the DL model classified one incomplete case as complete and five complete cases as incomplete. The distribution of errors reflects the substantial class imbalance inherent in this cohort and underscores the need for cautious interpretation of conventional accuracy and agreement metrics. A detailed failure case analysis of the clinically critical false-positive case is provided in Section 4.

A comprehensive summary of diagnostic performance metrics, including 95% CI, is provided in Table 3. The model achieved a sensitivity of 96.1% (95% CI, 92.2–98.9%) and a specificity of 85.7% (95% CI, 42.2–99.7%). Although the positive predictive value (PPV) was high at 99.2% (95% CI, 96.1–99.9%), the negative predictive value (NPV) was markedly lower (54.5%; 95% CI, 25.4–80.8%), reflecting the small number of incomplete cases. To account for the extreme prevalence imbalance, we additionally calculated the balanced accuracy, which was 90.8%. The F1-score was 0.976. The receiver operating characteristic (ROC) curve and precision recall curve (PRC) are presented in Figure 1. The ROC analysis yielded an AUC of 0.970 (95% CI, 0.929–1.000), while the area under the PRC for detecting incomplete placement was 0.727 with a 95% CI of 0.289–1.000, reflecting the small number of incomplete cases.

**Figure 2:**
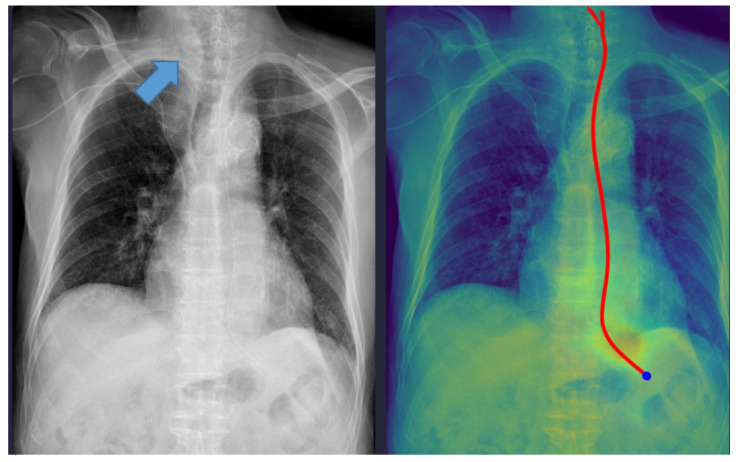
A misclassified incomplete nasogastric tube case identified as complete by the model. The arrow indicates the coiled portion of the nasogastric tube. The red line represents the trajectory of the nasogastric tube, and the blue dot indicates the tip of the nasogastric tube as identified by the deep learning model.

**Figure 3:**
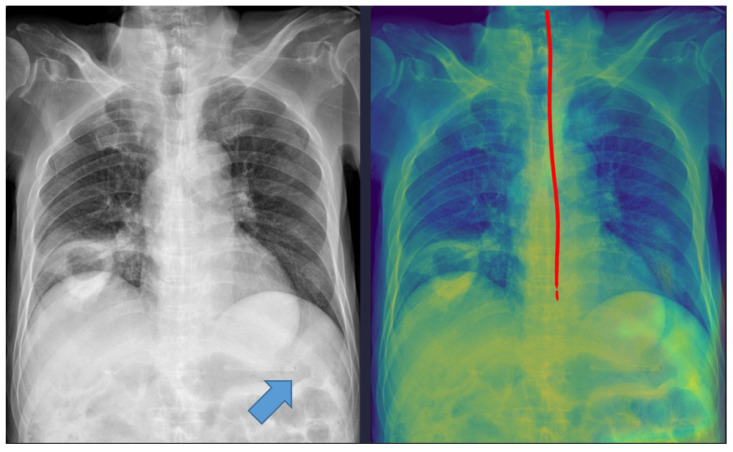
A misclassified complete nasogastric tube case identified as incomplete by the model. The arrow indicates the tip of the nasogastric tube. The red line represents the trajectory of the nasogastric tube as identified by the deep learning model.

**Figure 4:**
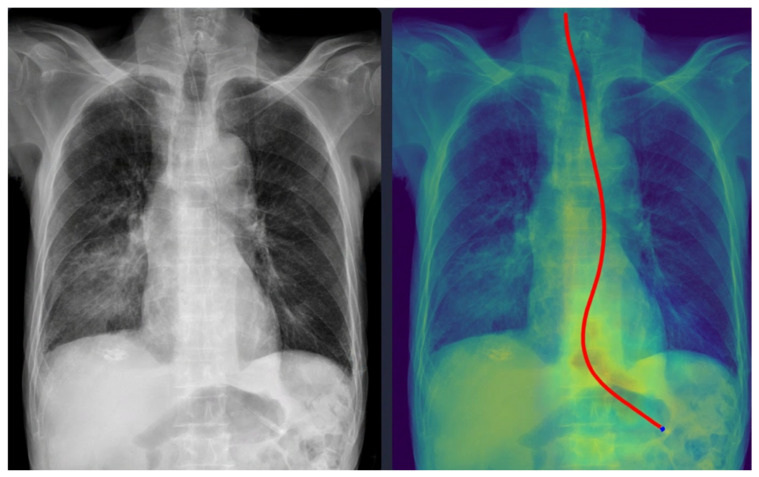
Correctly classified nasogastric tube case. The red line represents the trajectory of the nasogastric tube, and the blue dot indicates the tip of the nasogastric tube as identified by the deep learning model.

Representative examples of misclassified cases are shown in Figure 2 and Figure 3. The most critical failure involved an NG tube coiled at the indicated site in Figure 2, which suggested incomplete placement and a significant safety risk. The DL model misclassified this case as ‘complete’ based solely on the position of the tip projected below the gastroesophageal junction. Technically, this false-positive classification was highly likely due to the scarcity of such complex, coiled morphological features in the training dataset. While the segmentation module correctly identified the general tube structure, the dual-stage model failed to recognize this pronounced looping or coiling as an immediate risk factor, resulting in the classification module overriding the critical incomplete placement status. This finding identifies a crucial, previously unanticipated failure mode in the model’s design and carries substantial clinical significance, as initiating feeding through a coiled tube presents a high risk of aspiration pneumonia. Moving forward, efforts must focus on incorporating a greater diversity of these rare, yet clinically critical, coiled cases into the dataset to enhance the robustness of the tube segmentation process, thereby ensuring improved and safer clinical implementation. In Figure 3, the DL model failed to draw the entire trajectory of the NG tube and misclassified a complete case as incomplete. A representative example of a correctly classified case is shown in Figure 4. The tip of the NG tube is placed under the gastroesophageal junction and is safe to feed. Because most cases were correctly positioned (=95%), prevalence bias likely inflated the AC_1_ coefficient, and *κ* was lower despite near-identical classifications. Future studies should include more incomplete cases to obtain stable reliability estimates [12]. Prevalence-adjusted bias-adjusted kappa (PABAK) estimates were also evaluated with bootstrapping confidence intervals [13]. The results of the agreement among physicians and the DL model are shown in Table 4. Cohen’s κ showed the smallest estimate of 0.644 (95% confidence interval (CI): 0.366–0.922), which increased when using PABAK (0.911 (95% CI: 0.812–0.967)) and Gwet’s AC_1_ coefficient (0.956 (95% CI: 0.907–0.991)).

## 4. Discussion

NG tube insertions are among the most common procedures in critically ill patients [14], who are typically elderly and have a high prevalence of comorbidities [15]. A decreased serum albumin level is significantly associated with aging [16]. Due to these factors, patients requiring an NG tube for feeding are often elderly, have comorbidities such as chronic renal dysfunction and heart failure, and exhibit low serum albumin levels. These conditions can contribute to pulmonary edema, which makes it difficult to distinguish radiopaque devices, such as NG tubes, from surrounding structures. Misplacement or delayed recognition of NG tube position can cause aspiration pneumonia, esophageal perforation, or even fatal complications; therefore, rapid and reliable confirmation is crucial in critical care workflows [17].

As shown in Table 1, cases enrolled in this study were older adults, with a median age in the elderly range. Most patients were male, and the median BMI fell within the normal range for the Asian population. Laboratory values, including serum albumin, BUN, creatinine, and eGFR, reflected a population with chronic illness and reduced physiological reserve. When comparing correctly classified and misclassified cases, no statistically significant differences were observed for any clinical or radiographic variables. However, because the number of misclassified cases was extremely small (*n* = 6), the analysis was underpowered in detecting meaningful associations. Larger studies including more incomplete cases are required to determine whether specific patient or radiographic characteristics contribute to model failure.

The performance metrics in Table 3 demonstrate that the model reliably identified correctly positioned NG tubes but was less consistent in detecting incomplete placements. The high sensitivity and PPV were largely due to the predominance of complete cases; therefore, these values likely overestimate the true performance under balanced conditions. By contrast, the NPV and balanced accuracy were lower, indicating reduced stability in the minority class; curve-based metrics further highlighted this limitation. Although the AUC was higher (0.970) than that in a previous study which achieved an AUC of 0.90 (95% CI: 0.88–0.93) [3], its 95% CI (0.929–1.000) and the area under the RPC for incomplete detection (0.727, 95% CI: 0.289–1.000) reflect substantial uncertainty due to the very small number of incomplete cases. These findings suggest that the model’s performance for identifying malpositioned tubes remains insufficiently reliable, particularly in safety-critical scenarios. Overall, while the model shows promise for assisting in NG tube confirmation, it cannot be used as a standalone decision tool. Larger and more balanced datasets—especially with increased numbers of incomplete or malpositioned tubes—are required to better characterize model failures and to ensure safe clinical integration.

In this pilot evaluation, the DL model showed a high level of agreement with the physicians, achieving a Gwet’s AC_1_ coefficient of 0.956. However, these metrics must be interpreted with caution. Given the high prevalence of correctly positioned tubes in our dataset (approximately 95%), the reliability estimates may be influenced by the “prevalence paradox”, where agreement metrics can appear inflated. While the model demonstrated high specificity, the performance on the minority class (incomplete cases) highlights the necessity for further validation on balanced datasets.

The average time interval between NG tube insertion and chest radiograph acquisition was 31.5 min. The average time interval between radiograph acquisition and physician confirmation was 75 min, and in 13 cases (9.63%), the confirmation time exceeded 2 h. These time intervals were retrospectively extracted from the EMR. In a real-world clinical setting, nurses typically contact physicians after the radiograph is obtained, and the physicians subsequently confirm whether the NG tube can be used. Because of heavy clinical workloads, confirmation is frequently delayed. Previous studies reported that radiograph verification delayed NG tube use by ≥2 h in 51% of patients [2] and one study reported a mean confirmation time of 220 min [18].

When aided by the DL model, the average time required for the radiologist and pulmonologist to confirm NG tube placement was only 1.21 and 1.33 s, respectively. It is important to note that this “reading time” differs from the total “workflow turnaround time”. The DL model provides near-instantaneous interpretation support, which primarily targets the interpretation latency caused by physician unavailability rather than logistical delays. Therefore, these measurements do not imply a reduction in clinical confirmation time and were not considered part of the model’s performance assessment. Previous DL models for NG tube detection have primarily focused on classification [3,7,19]. By contrast, the model used in this study provides a visualization of the NG tube trajectory, facilitating identification of the tube tip. Integrating AI models directly into PACS or clinical decision-support platforms could streamline verification steps and reduce overall feeding delays, similar to prior studies demonstrating workflow acceleration through AI-assisted radiograph triage [20].

### 4.1. Failure Case Analysis and Safety Implications

Although the physicians agreed in all cases, six cases were discordant between the physicians and the DL model. One case was misclassified as ‘complete’ despite the NG tube being incompletely inserted (coiled), which could have been potentially fatal. As shown in Figure 2, the model interpreted it as complete because the tip of the NG tube was projected below the gastroesophageal junction level. The standalone Gradient-weighted Class Activation Mapping (Grad-CAM)-based heatmap and standalone segmentation-derived probability map are shown separately in Supplementary Figure S1. This figure demonstrates the interpretability of our model, which provides images of both maps overlaid together.

Technical and Clinical Analysis: Technically, this error likely stems from the models’ reliance on the vertical coordinate of the tube tip in the 2D projection. While the segmentation module correctly identified the tube structure, the classification head failed to recognize the “coiling” or “looping” morphological feature as a risk factor, likely due to the scarcity of such anomalous patterns in the training dataset. Clinically, initiating feeding through a coiled tube can lead to aspiration pneumonia. This failure mode highlights the inherent limitation of image-level classification models when dealing with complex 3D spatial configurations compressed into 2D images.

Safety Recommendations: To mitigate this risk, we emphasize that the DL model must strictly function as a “second reader” for trajectory visualization rather than a binary decision maker. In clinical practice, physicians must verify the Grad-CAM-based heatmap or segmentation mask overlaid on the original image. If the heatmap shows any deviation from a linear esophageal path (e.g., widening, looping), the placement must be flagged for manual review regardless of the high probability score. This aligns with the current paradigm of human–AI collaboration, where DL tools act as augmentative aids that enhance efficiency while maintaining physician oversight to ensure patient safety [21].

### 4.2. Limitations and Future Directions

Five cases were misclassified as incomplete, although the NG tube was correctly positioned in the stomach (Figure 3). In these cases, the model’s predicted pathway stopped midway, likely because radiopaque structures such as the spine overlapped with the tube. This finding indicates that the model requires additional training with a more diverse dataset to improve its robustness against anatomical noise.

Our study has several limitations. First, it was a retrospective single-center study with a relatively small sample size. Second, the dataset was collected from the same institution where a portion of the training data originated; therefore, the results represent an internal evaluation rather than full external validation. The generalizability of the model in applying images to the model from different X-ray vendors or different patient populations remains unproven. Third, the cases were highly imbalanced, with most showing correct NG tube placement, which limits the statistical power for evaluating sensitivity to misplacements. Future studies with larger, multi-center datasets that include a higher proportion of incorrect placements and diverse imaging equipment are warranted. Ultimately, explainable DL systems that provide real-time visual feedback may enhance both workflow efficiency and diagnostic confidence, contributing to safer and more timely enteral feeding in critical care practice.

## 5. Conclusions

In this pilot study, we evaluated a previously developed deep learning model for assessing NG tube placement on chest radiographs using real-world clinical data. The model showed strong performance in identifying correctly positioned tubes; however, its ability to detect incomplete placements was limited, as reflected by the low NPV, modest balanced accuracy, and the wide CI of the curve-based metrics. These findings highlight the instability of model performance in the minority class and underscore the challenges posed by the small number of incomplete cases.

Although the model may help streamline the confirmation process, physician oversight remains essential, particularly in safety-critical scenarios where misclassification could lead to patient harm. Therefore, the current model should be considered an assistive tool rather than a replacement for expert interpretation. Future work should include larger, multi-center datasets with more incomplete cases to better characterize model failure modes and establish more reliable, generalizable performance estimates.

## Figures and Tables

**Table 1:** Patient demographic characteristics.

Variables	Correctly Classified (*n* = 129)	Misclassified (*n* = 6)	*p*-Value
Age	77.32 ± 14.23	80.67 ± 16.44	0.423
Male	87 (67.44)	5 (83.33)	0.664
BMI (kg/m^2^)	21.89 ± 3.93	20.40 ± 3.88	0.650
WBC (/μL)	10,922.48 ± 10,070.56	10,433.33 ± 5079.24	0.729
Hemoglobin (g/dL)	10.95 ± 2.19	12.3 ± 3.26	0.367
Platelet (×10^3^/ μL)	207.73 ± 102.37	244.50 ± 92.33	0.384
Albumin (g/dL)	2.90 ± 0.59	3.15 ± 0.62	0.410
BUN (mg/dL)	26.97 ± 17.75	16.97 ± 8.85	0.160
Creatinine (mg/dL)	1.30 ± 1.21	0.69 ± 0.10	0.244
eGFR (mL/min/1.73 m^2^)	70.84 ± 31.72	92.67 ± 9.04	0.154
CT ratio	0.52 ± 0.08	0.48 ± 0.03	0.175
Medical devices	55 (42.64)	2 (33.33)	1

BMI, body mass index; WBC, white blood cell; BUN, blood urea nitrogen; eGFR, estimated glomerular filtration rate; CT, cardiac–thoracic.

**Table 2:** Confusion matrix comparing DL model predictions with physician-determined reference standard.

	Physicians	Complete	Incomplete
DL Model			
Complete		123	1
Incomplete		5	6

DL, deep learning.

**Table 3:** Final summary of diagnostic performance metrics of 95% confidence intervals.

Metric	Value	95% Confidence Interval
Sensitivity	0.961	0.922–0.989
Specificity	0.857	0.422–0.997
PPV	0.992	0.961–0.999
NPV	0.546	0.254–0.808
Balanced accuracy	0.909	
F1-score	0.976	

PPV, positive predictive value; NPV, negative predictive value.

**Table 4:** Estimates for Cohen’s κ, PABAK, and Gwet’s AC_1_ coefficient.

	Estimate (95% Confidence Interval)
Cohen’s κ	0.644 (0.366–0.922)
PABAK	0.911 (0.812–0.967)
Gwet’s AC_1_	0.956 (0.907–0.991)

PABAK, prevalence-adjusted bias-adjusted kappa.

## Data Availability

The datasets used in this study are not publicly available, due to patient privacy. However, de-identified data are available on request from the corresponding author, subject to approval by the Institutional Review Board of Kangwon National University Hospital.

## Supplementary Materials

The following supporting information can be downloaded at: https://www.mdpi.com/article/10.3390/tomography11120140/s1, Figure S1: Grad-CAM heatmap and segmentation/probability map of Figure 2; Table S1: Time intervals between NG tube insertion and physician confirmation

## Abbreviations

The following abbreviations are used in this manuscript:

NG	Nasogastric
DL	Deep learning
AUC	Area under the curve
AI	Artificial intelligence
BMI	Body mass index
PACS	Picture archiving and communication system
WBC	White blood cell
BUN	Blood urea nitrogen
eGFR	Estimated glomerular filtration rate
CT	Cardiac–thoracic ratio
EMR	Electronic medical records
PABAK	Prevalence-adjusted bias-adjusted kappa
CI	Confidence interval
PPV	Positive predictive value
NPV	Negative predictive value
ROC	Receiver operating characteristic
PRC	Precision–recall curve
Grad-CAM	Gradient-weighted class activation mapping
