# Supplementary material for: Pilot Evaluation of a Deep Learning Model for Nasogastric Tube Verification on Chest Radiographs: A Single-Center Retrospective Study
Source: Tomography. 2025 Dec 15;11(12):140. doi: 10.3390/tomography11120140 (PMC12737375; doi:10.3390/tomography11120140)
Supplement: Supplementary file 1 [file tomography-11-00140-s001.zip › tomography-3988366-supplementary.pdf]

Supplementary Table S1. Time intervals between NG tube insertion and physician confirmation

| | Time |
|---|---|
| Time interval between NG tube insertion and X-ray acquisition (min) | 31.50±52.29 |
| Time interval between X-ray acquisition and confirmation by doctors (min) | 75.60±111.44 |
| Reader 1 interpretation time assisted by the DL model (s) | 1.21±0.59 |
| Reader 2 interpretation time assisted by the DL model (s) | 1.33 ±0.37 |

NG, nasogastric; DL, deep learning; Reader 1, radiologist; Reader 2, pulmonologist

Supplementary Figure S1. Grad-CAM heatmap and segmentation/probability map of Figure 2

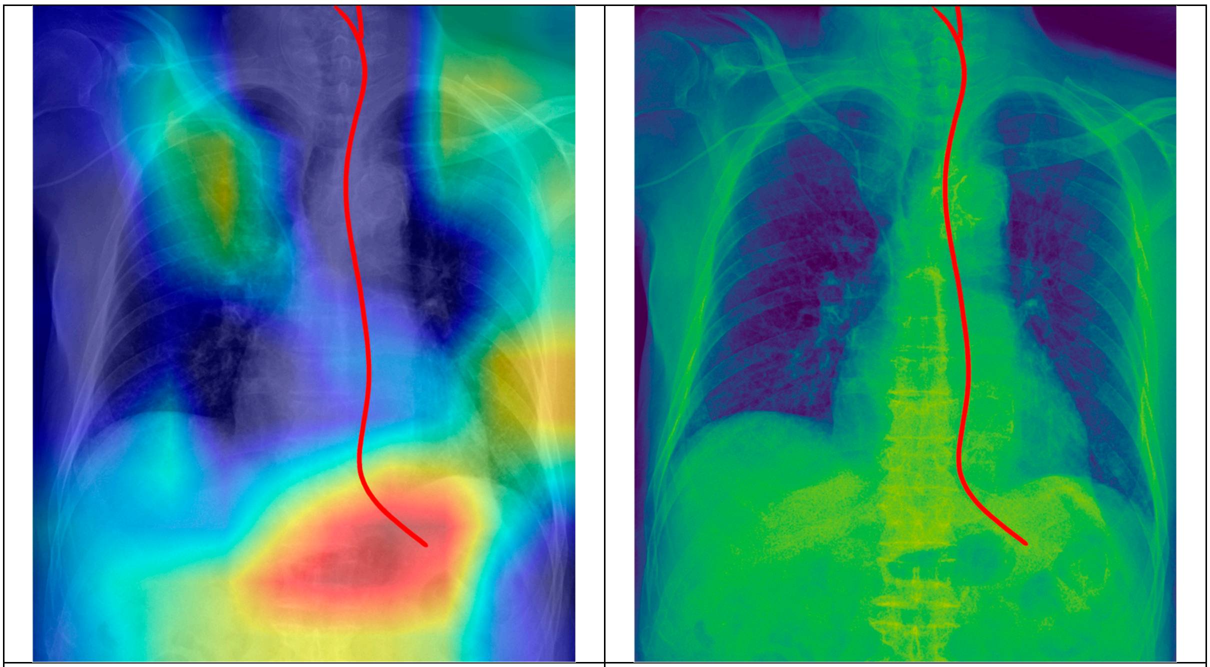

(a) Grad-CAM heatmap (b) Segmentation/probability map

Grad-CAM, Gradient-weighted class activation mapping
